# Primer design for the amplification of the ammonium transporter genes from the uncultured haptophyte algal species symbiotic with the marine nitrogen-fixing cyanobacterium UCYN-A1

**DOI:** 10.3389/fmicb.2023.1130695

**Published:** 2023-04-17

**Authors:** Krystal Salas, Ana M. Cabello, Kendra A. Turk-Kubo, Jonathan P. Zehr, Francisco M. Cornejo-Castillo

**Affiliations:** ^1^Department of Ocean Sciences, University of California, Santa Cruz, Santa Cruz, CA, United States; ^2^Centro Oceanográfico de Málaga, Instituto Español de Oceanografía, IEO-CSIC, Málaga, Spain; ^3^Department of Marine Biology and Oceanography, Institute of Marine Sciences (ICM-CSIC), Barcelona, Spain

**Keywords:** UCYN-A/haptophyte N2-fixing symbiosis, haptophyte biogeography, *Braarudosphaera bigelowii*, PCR primer design, ammonium transporter (*amt*) gene diversity

## Abstract

The multiple symbiotic partnerships between closely related species of the haptophyte algae *Braarudosphaera bigelowii* and the nitrogen-fixing cyanobacteria *Candidatus* Atelocyanobacterium thalassa (UCYN-A) contribute importantly to the nitrogen and carbon cycles in vast areas of the ocean. The diversity of the eukaryotic 18S rDNA phylogenetic gene marker has helped to identify some of these symbiotic haptophyte species, yet we still lack a genetic marker to assess its diversity at a finer scale. One of such genes is the ammonium transporter (*amt*) gene, which encodes the protein that might be involved in the uptake of ammonium from UCYN-A in these symbiotic haptophytes. Here, we designed three specific PCR primer sets targeting the *amt* gene of the haptophyte species (A1-Host) symbiotic with the open ocean UCYN-A1 sublineage, and tested them in samples collected from open ocean and near-shore environments. Regardless of the primer pair used at Station ALOHA, which is where UCYN-A1 is the pre-dominant UCYN-A sublineage, the most abundant *amt* amplicon sequence variant (ASV) was taxonomically classified as A1-Host. In addition, two out of the three PCR primer sets revealed the existence of closely-related divergent haptophyte *amt* ASVs (>95% nucleotide identity). These divergent *amt* ASVs had higher relative abundances than the haptophyte typically associated with UCYN-A1 in the Bering Sea, or co-occurred with the previously identified A1-Host in the Coral Sea, suggesting the presence of new diversity of closely-related A1-Hosts in polar and temperate waters. Therefore, our study reveals an overlooked diversity of haptophytes species with distinct biogeographic distributions partnering with UCYN-A, and provides new primers that will help to gain new knowledge of the UCYN-A/haptophyte symbiosis.

## Introduction

Biological dinitrogen (N_2_) fixation, i.e., the reduction of dissolved N_2_ gas into ammonia by N_2_-fixing prokaryotic microorganisms (diazotrophs), supplies new nitrogen (N) and thus supports primary production in the surface ocean (Zehr and Capone, 2020). In the ocean, some of these diazotrophs are symbiotic with phytoplankton species, which is the case for the group of unicellular N_2_-fixing symbiotic cyanobacteria known as *Candidatus* Atelocyanobacterium thalassa (hereafter UCYN-A). UCYN-A live in symbiosis with the unicellular haptophyte alga *Braarudosphaera bigelowii* and close relative species (Thompson et al., 2012; Hagino et al., 2013), being both symbiotic partners relevant contributors to marine N_2_ and carbon fixation, respectively, (Montoya et al., 2004; Martínez-Pérez et al., 2016; Zehr et al., 2016).

The diversity of the UCYN-A/haptophyte symbiosis has been explored using both phylogenetic and functional gene markers, which results have yielded differences in the number of phylotypes. For instance, while the 16S rDNA phylogenetic marker has resolved up to 3 distinct UCYN-A sublineages (UCYN-A1, −A2, and −A3) (Thompson et al., 2012; Hagino et al., 2013; Cornejo-Castillo et al., 2019), the diversity of the *nifH* gene, typically used for exploring the diversity of N_2_-fixers, has served to define up to 6–7 distinct UCYN-A sublineages with different biogeographical distributions (Farnelid et al., 2016; Turk-Kubo et al., 2017). However, regarding the diversity of the haptophyte species symbiotic with UCYN-A, only 2 different species have been identified based on the sequence diversity of the 18S rRNA phylogenetic marker (Thompson et al., 2012; Hagino et al., 2013; Cornejo-Castillo et al., 2019). Therefore, we currently lack a functional gene marker that could provide a finer phylogenetic resolution for these symbiotic haptophytes similar to the *nifH* gene marker in UCYN-A.

Not only is ammonium considered to be the preferred form of N for phytoplankton uptake (Glibert et al., 2016), but it has also been suggested to be one of the forms of N that these symbiotic haptophytes receive from UCYN-A (Sarkar et al., 2021). Ammonium addition experiments performed on natural populations of haptophyte species harboring UCYN-A1 or UCYN-A2 symbionts showed that these symbiotic haptophytes do not take up enough external ammonium to meet their nitrogen requirements, while UCYN-A kept providing fixed N to their haptophyte partners (Mills et al., 2020), further suggesting that UCYN-A is the main source of fixed N for these haptophytes. The protein involved in the uptake of ammonium is encoded by the ammonium transporter (*amt*) gene and, in metatranscriptomic data generated by the *TARA Oceans* project, this gene has been reported to be transcribed in the south Atlantic in, at least, the haptophyte species harboring the UCYN-A1 sublineage (Vorobev et al., 2020), altogether indicating that the *amt* gene might be relevant for the uptake of fixed N from UCYN-A in these haptophytes.

Here, we investigated the utility of the ammonium transporter (*amt*) gene as a meaningful marker to explore the diversity of the haptophyte species symbiotic with the UCYN-A1 sublineage at a fine-phylogenetic resolution. We designed three sets of PCR primer pairs to detect the *amt* gene of the haptophyte species (hereafter, A1-Host) that live in symbiosis with the nitrogen-fixing cyanobacterial sublineage UCYN-A1, which is considered to be the most abundant and widespread UCYN-A sublineage in open ocean waters (Cabello et al., 2016; Martínez-Pérez et al., 2016; Turk-Kubo et al., 2017). We tested these new PCR primer sets in environmental samples collected from a variety of locations, including both open ocean and near-shore sites (Table 1), and analyzed the amplified *amt* gene sequences. Our study offers a useful set of primers to detect these symbiotic haptophytes in environmental samples, and provides new insights on its biodiversity and biogeography.

**TABLE 1:** Sampling information for each of the samples used in this study.

Region	Sampling date	Latitude (degrees)	Longitude (degrees)	Volume filtered (L)	Sampling depth (m)	Size fraction (μm)	Pre-filter (μm)
North Pacific (Stn. 17 - Gradients2)	June, 2017	32.0	–158.0	3.15	25	0.2	No
North Pacific (Stn. ALOHA)	May, 2019	22.5	–158.0	2	25	0.2	No
South Pacific (Coral Sea)	Sep, 2012	–21.0	163.1	2.35	5	0.2	10
Arctic (Beaufort Sea)	July, 2009	70.56	–145.4	2	0	0.2	No
Arctic (Bering Sea)	Sep, 2016	64.7	–167.1	2	1	3	No
Tosa Bay (Kochi, Japan)	May, 2018	33.5	133.5	4	3	0.2	80
SIO Pier (San Diego, CA)	May, 2018	32.5	–117.2	1	2	0.2	150
California Current System (Baja California)	Oct, 2017	32.2	–118.9	2	50	0.2	210
Monterey Bay (Santa Cruz Wharf, CA)	June, 2017	36.96	–122.02	0.15	3	0.2	No

## Materials and methods

### Sampling information

The three new sets of *amt* PCR primer pairs designed here were tested in environmental DNA samples collected from diverse marine regions. Information about the geographic region and coordinates of the collected samples as well as the volume of seawater filtered, the size fraction collected, and whether the sample was pre-filtered for each sample is summarized in Table 1.

### Design of primers targeting the *amt* gene of the A1-Host

In order to design specific primers for the detection of the A1-Host *amt* gene, we first collected and analyzed the diversity of closely related *amt* gene sequences together with an A1-Host *amt* gene sequence (MATOU-v1_26947674) that had been identified as such based on co-abundant gene groups that included gene sequences from both UCYN-A1 and its haptophyte host (for further details see Vorobev et al., 2020). We performed a default blastn search on NCBI (nr/nt database; October 2019) using the A1-Host *amt* gene sequence (MATOU-v1_26947674) as query and collected *amt* gene sequences from different species (Table 2). In addition, we also included *amt* gene sequences from a study that explored the genetic structure of genes involved in the metabolism of N in phytoplankton species, including ammonium transporter genes (Bhadury et al., 2011).

**TABLE 2:** List of the *amt* gene sequences used to design primers.

Organism	GenBank (accession number)
A1-Host (MATOU-v1_26947674)	ERZ480625 (from ENA)
*Aureococcus anophagefferens*	XM_009034951.1
*Aureococcus anophagefferens*	GL833121.1
*Camellia sinensis*	AB117640.1
*Chlamydomonas reinhardtii*	XM_001693412.1
*Chlamydomonas reinhardtii*	XM_001697452.1
*Chlamydomonas reinhardtii*	DS496109.1
*Cylindrotheca fusiformis*	AF360394.1
*Cylindrotheca fusiformis*	AY651853.1
*Dunaliella viridis*	GU592656.1
*Ectocarpus siliculosus*	FN648668.1
*Ectocarpus siliculosus*	FN648387.1
*Emiiana huxleyi* CCMP1516	XM_005785291.1
*Emiiana huxleyi* CCMP1516	XM_005777257.1
*Micromonas commoda*	XM_002508364.1
*Micromonas pusilla* CCMP1545	GG663747.1
*Micromonas* sp. RCC299	CP001574.1
*Micromonas* sp. RCC299	CP001576.1
*Monoraphidium neglectum*	XM_014042203.1
*Myxococcus xanthus*	CP000113.1
*Ostreococcus tauri*	CAID01000020.1
*Perkinsus marinus*	GG680729.1
*Phaeodactylum tricornutum*	CM000622.1
*Phaeodactylum tricornutum*	CP001141.1
*Rhodothermus marinus*	CP001807.1
*Sorghum bicolor*	JX294852.1
*Tetraselmis chuii*	JN807467.1
*Thalassiosira pseudonana*	CM000639.1
*Triticum aestivum*	AY525637.3
Uncultured marine eukaryote clone S04GAmt22	JN807524.1
Uncultured marine eukaryote clone S08GAmt33	JN807540.1

The first sequence shows the A1-Host *amt* gene, while the following rows show the collection of *amt* gene sequences from diverse organisms that we used to ensure the specificity of the primers toward the A1-Host. GenBank accession numbers are indicated in the second column.

We used the *amt* gene sequence diversity to identify unique regions within the A1-Host *amt* gene sequence. Briefly, *amt* gene sequences were aligned using the multiple sequence alignment (MSA) tool in Geneious Software (11.0.5). Regions of the *amt* sequence with sufficient mismatches across species to ensure specificity of amplification of the primers toward A1-Host were carefully selected and three different primer pairs targeting different regions of the A1-Host *amt* gene sequence were designed (Table 3). In addition, to further assure the specificity *in silico* of the newly designed primers, we ran the ‘Primer BLAST’ tool through NCBI.

**TABLE 3:** Forward and reverse primer sequences for each of the designed primer pairs.

Forward primer		Reverse primer	
**Name**	**Sequence (5′ -> 3′)**	**Name**	**Sequence (5′ -> 3′)**
AMT-555F	CGTTGTGCACATGACTGG	AMT-705R	GACCCAGAGGATGAAGGTTC
AMT-557F	TTGTGCACATGACTGGCGGT	AMT-715R	CGTACCAGCCGACCCAGA
AMT-330F	GTTCTTCCAGTTCGTCTTCGCT	AMT-456R	GACGACAGGGTACACGAAAG

### DNA extraction and PCR amplification

DNA extractions were performed using a protocol based on the DNeasy Plant Mini kit (Qiagen) with slight modifications following Moisander et al. (2008). DNA extracts were stored at −80°C. The following PCR program was used: initial denaturation at 95°C for 3 min, followed by 25 cycles of denaturation at 95°C for 30 s, annealing at 58°C for 30 s and elongation at 72°C for 30 s. All PCR reactions (25 μL) contained Platinum*™* Taq DNA polymerase (6 units; Invitrogen, Carlsbad, CA, USA), 1X PCR Buffer (-MgCl_2_), 4 mM MgCl2, 400 μM dNTP mix, 0.5 μM of each primer (forward and reverse) (Table 3), and 1 μL of template DNA. PCR products were visualized by electrophoresis on 1% agarose gel using 1X TBE buffer diluted with milliQ and 100BP Gene Ladder (Thermo Scientific GeneRuler 100 bp Gene Ladder).

### Illumina sequencing

The PCR amplification used the newly designed primers (Table 2) with 5′ common sequence linkers CS-F (5′-ACA CTG ACG ACA TGG TTC TAC A-3′) in the forward primers and CS-R (5′-TAC GGT AGC AGA GAC TTG GTC T-3′) in the reverse primers (Moonsamy et al., 2013). Reaction conditions and thermocycling parameters are described in the previous section. Library preparation and amplicon sequencing using Illumina MiSeq technology was carried out at the DNA Services Facility at the University of Chicago, Illinois.^1^ A total of 1,365,483 raw paired-end reads were obtained, ranging from 27,278 up to 118,887 read counts per sample (integrating two technical replicates per sample) for a total of 19 samples (Supplementary Table 1).

### Ammonium transporter (*amt*) gene sequence analysis

The dada2 pipeline was used for processing and analyzing the amplicon data of the Illumina sequences following the protocol published in https://benjjneb.github.io/dada2/index.html (Callahan et al., 2016; Supplementary Table 1). In order to determine amplicon sequence variants (ASVs) closely related to the A1-Host *amt* gene, those ASVs accounting for >0.5% of the total sequences were aligned with the A1-Host *amt* gene using the MSA Tool in Geneious Software (11.0.5). Sequences sharing at least 95% identity at the nucleotide level with the A1-Host *amt* gene were classified as A1-Host closely related ASVs since this percentage has been previously defined as a convenient threshold to co-assemble single-amplified protist genomes that belonged to the same species (Latorre et al., 2021).

## Results

### Design of PCR primers targeting the A1-Host ammonium transporter (*amt*) gene

In order to detect and amplify the A1-Host *amt* gene from environmental samples, three primer pairs (AMT-555F/AMT-705R, AMT-557F/AMT-715R, and AMT-330F/AMT-456R) targeting different regions of the A1-Host *amt* gene sequence were designed (Table 3). Briefly, after aligning the A1-Host *amt* gene sequence with closely related *amt* sequences, we detected three conserved nucleotide regions with enough mismatches that would potentially ensure specificity of the primers toward the A1-Host *amt* gene (Supplementary Figures 1–3). The primer pair AMT-555F/AMT-705R showed at least 3 mismatches (2 and 1 with AMT-555F and AMT-705R, respectively) with non-A1-Host *amt* gene sequences (Supplementary Figure 1). Similarly, the primer pair AMT-557F/AMT-715R showed at least 3 mismatches (2 and 1 with AMT-557F and AMT-715R, respectively) with non-A1-Host *amt* gene sequences (Supplementary Figure 2). Primers AMT-330F/AMT-456R showed at least 6 mismatches (2 and 4 with AMT-330F and AMT-456R, respectively) with non-A1-Host *amt* gene sequences (Supplementary Figure 3).

### Performance of A1-Host *amt* PCR primers in environmental samples

To test the performance of the primers for amplifying the A1-Host *amt* gene sequence, PCR amplifications using each of the three different *amt* PCR primer sets were carried out using environmental samples collected from open ocean [North Pacific, Coral Sea (South Pacific), Arctic Ocean (Bering Sea and Beaufort Sea)] and near-shore locations [Tosa Bay (Japan), Santa Cruz (CA, USA) and San Diego (CA, USA)] (Table 1 and Supplementary Table 1). These samples were chosen because they had previously been shown to contain different UCYN-A sublineages based on *nifH* gene sequencing and/or Catalyzed Reporter Deposition Fluorescence *In Situ* Hybridization (CARD-FISH) (Hagino et al., 2013; Harding et al., 2018; Henke et al., 2018; Cornejo-Castillo et al., 2019; Cabello et al., 2020; Gradoville et al., 2020; Turk-Kubo et al., 2021). PCR products showed that primers AMT-557F/AMT-715R amplified DNA fragments of the expected size in all samples except the Santa Cruz Wharf (Supplementary Figure 4). Likewise, primers AMT-555F/AMT-705R showed amplification in the North Pacific and Arctic Ocean samples, although dim PCR bands could also be observed in the Coral Sea and Tosa Bay (Japan) (Supplementary Figure 4). Finally, the AMT-330F/AMT-456R primer pair showed a more restricted amplification, only exhibiting PCR bands in the North Pacific and Coral Sea samples (Supplementary Figure 4).

**FIGURE 1:**
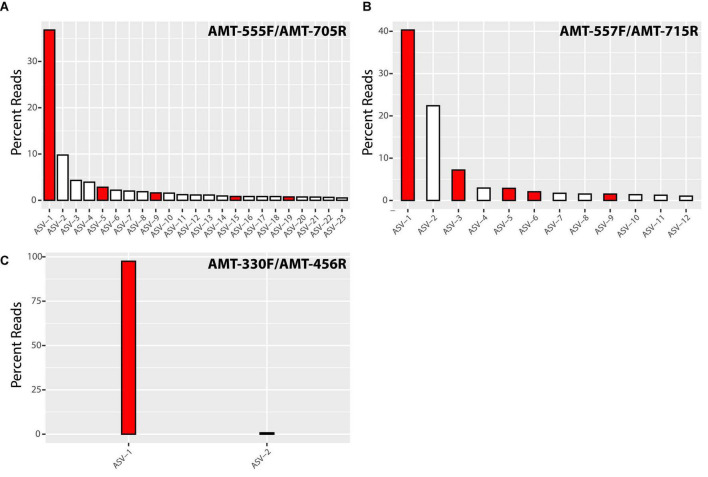
Bar chart of the percentage of total reads represented by significant amplicon sequence variants (ASVs) amplified by AMT-555F/AMT-705R **(A)**, AMT-557F/AMT-715R **(B)** and AMT-330F/AMT-456R **(C)** primer pairs. We have defined significant ASVs as those accounting for more than 0.5% of total reads. Red bars represent ASVs closely related (>95% nucleotide identity) to the A1-Host *amt* gene, while white bars indicate non-A1-Host related ASVs.

**FIGURE 2:**
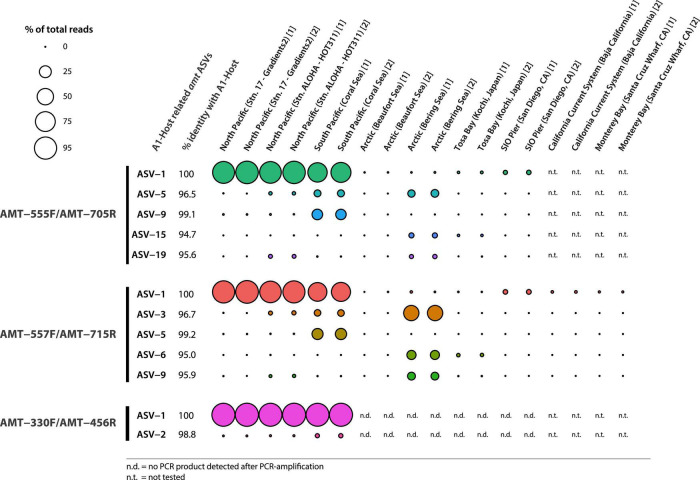
Bubble chart representing the relative abundance of ASVs closely related to the A1-Host *amt* gene (red bars in Figure 1) across samples (technical replicates are indicated with numbers between brackets after the name of the samples). ASVs amplified by different primer pairs are indicated. Bubble size is determined by percent reads represented by every ASV within the same sample. Closely related ASVs and the percent identity shared with the A1-Host *amt* gene are shown as well as the % of reads captured in each of the tested samples.

Illumina sequencing of the PCR products showed the diversity of *amt* gene sequences captured by each *amt* PCR primer set. In total, the primer pairs AMT-555F/AMT-705R and AMT-557F/AMT-715R recovered a higher number of amplicon sequence variants (ASVs) across samples than the AMT-330F/AMT-456R primer pair (Figure 1). In particular, the primer pair AMT-555F/AMT-705R generated 596 ASVs, and similarly, the primer pair AMT-557F/AMT-715R amplified 733 ASVs. ASVs captured by these primer pairs and accounting for greater than 0.5% of the total reads, represented 23 ASVs and 78% of the total reads for the primer pair AMT-555F/AMT-705R, and 12 ASVs and 86% of the reads for the primer pair AMT-557F/AMT-715R (Figure 1). The most abundant ASV, i.e., the one containing the highest number of reads, for both the AMT-555F/AMT-705R [ASV-1_(555F/705R)_] and the AMT-557F/AMT-715R [ASV-1_(557F/715R)_] primer pairs shared 100% nucleotide identity with the A1-host *amt* gene (Figure 2). ASV-1_(555F/705R)_ and ASV-1_(557F/715R)_ were mainly found in the North Pacific and Coral Sea, and also at the SIO Pier (San Diego) but at much lower relative abundance (Figure 2). Other ASVs showing high percentage of similarity with the A1-Host *amt* gene were recovered with the AMT-555F/AMT-705R primer pair, specifically ASV-5_(555F/705R)_, ASV-9_(555F/705R)_, ASV-15_(555F/705R)_, and ASV-19_(555F/705R)_ shared 96.5, 99.1, 94.7, and 95.6% nucleotide identity with the A1-Host *amt* gene, respectively, (Figure 2). Interestingly, these ASVs had a different distribution than the ASV-1_(555F/705R)_, for instance, ASV-9_(555F/705R)_, found preferentially in the Coral Sea, or ASV-19_(555F/705R)_, in the Bering Sea (Figure 2).

The primer pair AMT-557F/AMT-715R captured mostly the same diversity as the primer pair AMT-555F/AMT-705R (Figure 2). Therefore, the primer pair AMT-557F/AMT-715R, in addition to ASV-1_(557F/715R)_, also captured ASVs that were closely related to the A1-Host *amt* gene. In particular, ASV-3_(557F/715R)_, ASV-5_(557F/715R)_, ASV-6_(557F/715R)_, and ASV-9_(557F/715R)_ shared 96.7, 99.2, 95.0, and 95.9% nucleotide identity with the A1-Host *amt* gene, respectively, (Figure 2). As shown before, these ASVs were also closely related to the A1-Host, and yet showed a different distribution than ASV-1_(557F/715R)_. For instance, ASV-3_(557F/715R)_, ASV-6_(557F/715R)_, and ASV-9_(557F/715R)_ were relatively more abundant than ASV-1_(557F/715R)_ in the Bering Sea, and ASV-3_(557F/715R)_ also was present in the Coral Sea and North Pacific with lower relative abundance when compared with ASV-1_(557F/715R)_ (Figure 2).

In contrast, the primer pair AMT-330F/AMT-456R amplified a much lower diversity of *amt* gene sequences, capturing a total of 103 ASVs. More than 98% of the total reads clustered in a single ASV [ASV-1_(330F/456R)_] that shared 100% nucleotide identity with the A1-Host *amt* gene (Figure 2). ASV-1_(330F/456R)_ was the dominant variant in the North Pacific and the Coral Sea samples (Figure 2). The only one additional ASV with a relative abundance above 0.5% was ASV-2_(330F/456R)_, and shared a very high percentage of nucleotide identity (98.8%) with the A1-Host *amt* gene. ASV-2_(330F/456R)_ accounted for 0.8% of total reads and was mainly found in the Coral Sea, and also at lower relative abundance in the North Pacific samples (Figure 2). It is worth noticing that using the AMT-330F/AMT-456R primer pair, no PCR products were amplified in samples from the Arctic Ocean, Tosa Bay (Japan) or SIO Pier (San Diego, CA) (Supplementary Figure 4).

## Discussion

In this study, we designed and tested new PCR primers to detect and explore the diversity of *amt* gene sequences of the haptophytes species symbiotic with the UCYN-A1 sublineage (A1-Host). The three PCR primer sets showed preferential amplification toward the A1-Host *amt* gene (ASV-1), in particular in samples where UCYN-A1 is the UCYN-A dominating sublineage, such as in the North Pacific and Coral Sea (Turk-Kubo et al., 2017; Henke et al., 2018; Cornejo-Castillo et al., 2019; Gradoville et al., 2020; Figure 2). The primer pair AMT-330F/AMT-456R showed the highest specificity to amplify the A1-Host *amt* gene, which was further confirmed by the lack of PCR amplification product in samples collected at the locations typically associated with UCYN-A2, such as San Diego (SIO Pier), Baja California, Tosa Bay (Japan), and Santa Cruz (Santa Cruz Municipal Wharf) (Hagino et al., 2013; Bombar et al., 2014; Thompson et al., 2014; Turk-Kubo et al., 2017; Cornejo-Castillo et al., 2019; Cabello et al., 2020), thus suggesting that this primer set does not amplify the *amt* gene of haptophytes partnering with the UCYN-A2 sublineage (Thompson et al., 2014).

The AMT-555F/AMT-705R and the AMT-557F/AMT-715R primer sets both presented a similar amplification pattern across locations and provided a higher number of ASVs than the primer pair AMT-330F/AMT-456R, revealing new *amt* genetic diversity (Figure 2). The A1-Host *amt* gene (ASV-1) was detected at the SIO Pier (San Diego, CA), although at very low relative abundances using the AMT-555F/AMT-705R and the AMT-557F/AMT-715R primer pairs, but not with the AMT-330F/AMT-456R primer pair. The detection of the A1-Host *amt* gene is consistent with previous studies showing the presence of the UCYN-A1 sublineage in low abundance in UCYN-A2-dominated coastal regions (Turk-Kubo et al., 2017; Cornejo-Castillo et al., 2019). However, the lack of recovery of the ASV-1 in these coastal areas when using the AMT-330F/AMT-456R primer pair could reflect slightly divergent *amt* sequences of A1-Host ecotypes adapted to different environments (oligotrophic subtropical versus near-shore areas). Similarly, although the UCYN-A1 sublineage has been previously detected in the Arctic (Harding et al., 2018; Shiozaki et al., 2018), the most abundant A1-Host related ASV amplified with the primer pairs AMT-555F/AMT-705R [ASV-1_(555F/705R)_] and AMT-557F/AMT-715R [ASV-1_(557F/715R)_] exhibited little to no amplification in those same Arctic samples.

The second most abundant *amt* host-related ASV detected with the primers AMT-555F/AMT-705R [ASV-5_(555F/705R)_] and AMT-557F/AMT-715R [ASV-3_(557F/715R)_], showed amplification not only in Arctic samples but also in the Coral Sea and Pacific Ocean, suggesting the presence of a divergent A1-Host that might be in symbiosis with a closely related (but different) UCYN-A1 sublineage in those areas. Also, both primer pairs AMT-555F/705R and AMT-557F/AMT-715R amplified ASVs that share ca. 95% of nucleotide identity with the A1-Host in the North and South Pacific regions, where UCYN-A3 has been previously reported (Thompson et al., 2014; Henke et al., 2018; Cornejo-Castillo et al., 2019), which suggests that some of the amplified ASVs might correspond with the *amt* gene of the haptophyte species partnering with the UCYN-A3 sublineage. Additionally, the biogeographic distribution of ASV-5_(555F/705R),_ ASV-9_(555F/705R),_ ASV-3_(557F/715R)_, and ASV-5_(557F/715R)_ was limited to the Coral Sea, where a genetic variant of the UCYN-A2 sublineage (oligo43) has been previously found (Turk-Kubo et al., 2017). Due to their genetic closeness, we hypothesize that some of these amplified ASVs might represent new *amt* gene diversity of haptophytes partnering divergent UCYN-A sublineages, although further investigations will be needed to verify this hypothesis.

Although all the non-target sequences (i.e., sequences from species *a priori* not associated symbiotically with UCYN-A) that were considered during the design of the *amt* primers presented mismatches with the primer sequences (Supplementary Figures 1–3), these might be not enough to prevent the amplification of some of them. For instance, when considering all samples together, the primers AMT-555F/AMT-705R amplified non-A1-Host ASVs that accounted for 35% of the total relative abundance (Figure 1). However, this percentage comes mainly from samples where the A1-Host was absent (or in a very low relative abundance) such as the Arctic samples (Figure 2), suggesting that the amplification of non-target sequences could be more important in samples where the A1-Host and/or its close-relative species are not present. Similarly, when using the primer pair AMT-557F/AMT-715R, non-A1-Host ASVs accounted for 32% of the total relative abundance (Figure 1) in samples where the A1-Host and/or its close-relative species were mainly absent, such as in the Santa Cruz Wharf, Baja California, Beaufort Sea, and Tosa Bay (Japan) (Figure 2). These are locations where UCYN-A2 dominates over UCYN-A1 (Cabello et al., 2020), which supports the hypothesis that both AMT-555F/AMT-705R and AMT-557F/AMT-715R primer pairs might capture non-target *amt* diversity when the A1-Host is in low abundance (or absent). Finally, either the lack of PCR amplification (Supplementary Figure 3) or the absence of dominant ASVs related with (but not identical to) the A1-Host *amt* gene in locations where UCYN-A2 dominates over UCYN-A1 suggests that none of the three newly designed primer pairs would amplify the *amt* gene of the UCYN-A2 host.

## Conclusion

Our study offers new PCR primers for the detection of the *amt* gene of haptophytes species symbiotic with UCYN-A1, key members of the C and N cycles in the open ocean. The biogeography of the *amt* gene sequence variants shown in this study were in agreement with previous findings based on the UCYN-A1 *nifH* marker gene and CARD-FISH research, thus validating the use of these novel *amt*-targeted PCR primers as a proxy to explore the distribution of the UCYN-A1/haptophyte symbiosis. Furthermore, these new primers uncovered new variants of the *amt* gene that might represent an overlooked diversity of haptophytes harboring UCYN-A symbionts with distinct biogeographical distributions that needs to be further explored in future studies.

## Data availability statement

The sequencing data presented in this study are deposited in the NCBI repository, under the accession number: PRJNA946153 (https://www.ncbi.nlm.nih.gov/sra/PRJNA946153).

## Author contributions

FC-C and JZ conceptualized the study. KS designed the PCR primers with advice from FC-C and tested the primers in environmental samples with the help of AC and KT-K. KS and FC-C analyzed the sequencing data and prepared the manuscript with contributions of all co-authors. All authors contributed to the article and approved the submitted version.

## Footnotes

1
rrc.uic.edu/cores/genome-research/sequencing-core/
