# Supplementary material for: Primer design for the amplification of the ammonium transporter genes from the uncultured haptophyte algal species symbiotic with the marine nitrogen-fixing cyanobacterium UCYN-A1
Source: Front Microbiol. 2023 Apr 17;14:1130695. doi: 10.3389/fmicb.2023.1130695 (PMC10150950; doi:10.3389/fmicb.2023.1130695)

Supplementary figures and tables

Suppl. Table 1. Summary of reads remaining after each quality check step of the dada2 pipeline. The sample identifier for technical replicates (sample ID), locations associated with each sample, accession numbers, initial raw reads (input), reads remaining after standard filtering process (filtered), reads remaining after denoising forward (denoisedF) and reversed (denoisedR) reads, merged reads after previous quality checks (merged) and final reads after detecting and removing chimeric (nonchim) are indicated.

Primer pair	Sample location [technical replicate #]	Accession #	input	filtered	denoisedF	denoisedR	merged	nonchim
AMT-557F/AMT-715R	Monterey Bay (Santa Cruz Wharf, CA)[1]	SAMN33813769	6810	6675	5821	5675	929	929
	Monterey Bay (Santa Cruz Wharf, CA)[2]	SAMN33813770	32451	31638	31154	31398	5162	5162
	California Current System (Baja California)[1]	SAMN33813771	14779	14597	13145	13087	9911	9911
	California Current System (Baja California)[2]	SAMN33813772	59062	58464	57897	58274	38425	38389
	North Pacific (Stn. ALOHA - HOT311)[1]	SAMN33813773	18271	18007	16154	16334	12205	12205
	North Pacific (Stn. ALOHA - HOT311)[2]	SAMN33813774	75148	74475	73759	74209	49487	49474
	Arctic (Beaufort Sea)[1]	SAMN33813775	8380	8186	6821	6816	3596	3596
	Arctic (Beaufort Sea)[2]	SAMN33813776	31434	31063	30365	30833	15936	15936
	SIO Pier (San Diego, CA)[1]	SAMN33813777	12214	12040	10424	10383	8263	8226
	SIO Pier (San Diego, CA)[2]	SAMN33813778	55251	54653	53659	54186	34077	34000
	Arctic (Bering Sea)[1]	SAMN33813779	19033	18719	16434	16583	12836	12455
	Arctic (Bering Sea)[2]	SAMN33813780	78394	77691	76256	76794	50515	50177
	Tosa Bay (Kochi, Japan)[1]	SAMN33813781	12710	12517	10986	10985	8035	8011
	Tosa Bay (Kochi, Japan)[2]	SAMN33813782	60235	59538	58814	59131	36324	36232
	South Pacific (Coral Sea)[1]	SAMN33813783	16605	16381	14730	14912	11660	11585
	South Pacific (Coral Sea)[2]	SAMN33813784	70757	70082	69691	69992	46936	46844
	Pacific Ocean (Stn. 17 Gradients2)[1]	SAMN33813785	100397	99602	98955	99349	67174	67174
	Pacific Ocean (Stn. 17 Gradients2)[2]	SAMN33813786	23144	22773	20950	21106	16051	16051
AMT-555F/AMT-705R	North Pacific (Stn. ALOHA - HOT311)[1]	SAMN33813787	14407	14168	12916	13017	10779	10779
	North Pacific (Stn. ALOHA - HOT311)[2]	SAMN33813788	32729	32299	31943	31961	30419	30419
	Arctic (Beaufort Sea)[1]	SAMN33813789	17496	17246	16229	16222	13876	12269
	Arctic (Beaufort Sea)[2]	SAMN33813790	36867	36395	36118	36174	34528	32028
	SIO Pier (San Diego, CA)[1]	SAMN33813791	6454	6344	5766	5789	5450	5280
	SIO Pier (San Diego, CA)[2]	SAMN33813792	20824	20457	20059	20096	19134	18751
	Arctic (Bering Sea)[1]	SAMN33813793	24663	24369	22561	22650	19045	17647
	Arctic (Bering Sea)[2]	SAMN33813794	56721	55865	55200	55455	53016	51600
	Tosa Bay (Kochi, Japan)[1]	SAMN33813795	11653	11471	10489	10580	9319	9319
	Tosa Bay (Kochi, Japan)[2]	SAMN33813796	27840	27336	26908	27071	25853	25851
	South Pacific (Coral Sea)[1]	SAMN33813797	6073	5975	5556	5560	4651	4559
	South Pacific (Coral Sea)[2]	SAMN33813798	22597	22287	21974	22067	21018	21018
	Pacific Ocean (Stn. 17 Gradients2)[1]	SAMN33813799	21867	21566	20051	20173	16323	16323
	Pacific Ocean (Stn. 17 Gradients2)[2]	SAMN33813800	44208	43655	43419	43494	41779	41777
AMT-330F/AMT-456R	North Pacific (Stn. ALOHA - HOT311)[1]	SAMN33813801	19496	19070	16829	17073	12511	12511
	North Pacific (Stn. ALOHA - HOT311)[2]	SAMN33813802	80221	79415	78732	79200	49855	49855
	South Pacific (Coral Sea)[1]	SAMN33813803	23531	22951	20552	20075	12411	12411
	South Pacific (Coral Sea)[2]	SAMN33813804	83874	83215	82975	83092	52517	52505
	Pacific Ocean (Stn. 17 Gradients2)[1]	SAMN33813805	21482	21130	19170	19186	14471	14471
	Pacific Ocean (Stn. 17 Gradients2)[2]	SAMN33813806	97405	96620	96010	96357	60884	60880

Supplementary Figure 1. Priming site of the newly designed AMT-555F/AMT-705R primers. The first row shows the priming site within the A1-Host *amt* gene. Following rows show the priming sites within the closely related *amt* sequences used to design the primers. Priming sites with gaps in the associated gene sequences are excluded from the figure. Mismatches in the gene sequences are shown in bolded black text. The end positions for the priming site within the A1-Host *amt* gene sequence are labeled accordingly. Each sequence is written in 5' to 3' orientation.

Organism	Accession ID	AMT-555F priming site (555–572)	AMT-705R priming site (686–705)
		C G T T G T G C A C A T G A C T G G	G A A C C T T C A T C C T C T G G G T C
A1-Host	MATOU-v1_26947674	C G T T G T G C A C A T G A C T G G	G A A C C T T C A T C C T C T G G G T C
Chlamydomonas reinhardtii	XM_001697452.1	C G T C G T G C A C A T G A C G G G	G C A C C T T C C T G T T G T G G T T C
Chlamydomonas reinhardtii	XM_001693412.1	C G T C G T G C A C A T G G T C G G	G C A C G T T C C T C C T C T G G G T C
Aureococcus anophagefferens	XM_009034951.1	C G T C G T G C A C A T G A C C G G	G C A C C T T C A T C C T C T G G G T C
Micromonas commoda	XM_002508364.1	C G T C G T G C A C A T G A C T G G	G C A C C G T G C T G C T G T G G T T C
Emiliania huxleyi CCMP1516	XM_005785291.1	C G T G G T G C A C T T G A C A G G	G C A C C T T C A T C C T C T G G A T G
Emiliania huxleyi CCMP1516	XM_005777257.1	C G T G G T G C A C T T G A C G G G	G C A C C T T C A T T C T C T G G A T G
Monoraphidium neglectum	XM_014042203.1	C G T C G T G C A C A T G G T G G G	G C A C G G T C C T G C T C T G G T T C
Dunaliella viridis	GU592656.1	T G T T G T C C A C A T G A C G G G	G T A C C C T C A T C C T G T G G T T C
Sorghum bicolor	JX294852.1	C G T C G T C C A C A T G G T C G G	G C A C C T T C C T G C T C T G G T T C
Triticum aestivum	AY525637.3	G G T T G T C C A C A T G G T C G G	G C A C C T T C T T G C T G T G G T T T
Micromonas pusilla CCMP1545	GG663747.1	C G T C G T T C A C A T G G T C G G	G C A C C T T C A T C C T C T G G G T T
Ectocarpus siliculosus	FN648668.1	C G T C G T C C A C A T G A C C G G	G C A C G C T C A T C C T C T G G T T C
Ectocarpus siliculosus	FN648387.1	C G T C G T C C A C A T G A C C G G	G C A C G C T C A T C C T C T G G T T C
Ostreococcus tauri	CAID01000020.1	T A T C G T C C A C A T G A C T G G	G C A C C T T C A T C C T C T G G C T C
Cylindrotheca fusiformis	AF360394.1	T G G A G C - - - C A C T G C T C T	G T A C C T T C C T T C T T T G G T T C
Chlamydomonas reinhardtii	DS496109.1	C G T C G T G C A C A T G A C T G G	G C A C C G T G C T G C T G T G G T T C
Camellia sinensis	AB117640.1	A G T C G T A C A C A T G G T C G G	G A A C G T T C C T T C T A T G G T T C
Aureococcus anophagefferens	GL833121.1	C G T C G T G C A C A T G G T C G G	G C A C G T T C C T C C T C T G G G T C
Myxococcus xanthus	CP000113.1	G G T G G T G C A C A T G T C C G C	G C A C G G G C A T G C T G T G G T T C
Micromonas sp. RCC299	CP001574.1	A A T C G T C C A T A T G A C A G G	G A A C C T T T A T T C T A T G G T T C
Rhodothermus marinus	CP001807.1	C G T G G T G C A T G C C G T C G G	G C G T C T T C A T C C T C T T C T T C
Micromonas sp. RCC299	CP001576.1	C G T C G T G C A C A T G A C C G G	G C A C C T T C A T C C T C T G G G T C
Perkinsus marinus	GG680729.1	T A T T G T C C A T C T T A C C G G	G T A C T T T T G T T C T G T G G T T C
Phaeodactylum tricornutum	CM000622.1	A C C A G T C C A C A T G A C C G G	G A A C G T T T T G T C T T T G G T T T
Phaeodactylum tricornutum	CP001141.1	A G T A G T G C A C A T G A C T G G	G T A C C T T C A T T C T C T G G T T T
Cylindrotheca fusiformis	AY651853.1	T G T T G T G C A T A T G T G C G G	G T A C C T T C C T T C T T T G G T T C
Thalassiosira pseudonana	CM000639.1	A C C A G T T C A C A T G T G T G G	G A A C C T T T G C T C T T T G G T T C

Supplementary Figure 2. Priming site of the newly designed AMT-557F/AMT-715R primers. The first row shows the priming site within the A1-Host *amt* gene. Following rows show the priming sites within the closely related amt sequences used to design the primers. Priming sites with gaps in the associated gene sequences are excluded from the figure. Mismatches in the gene sequences are shown in bolded black text. The end positions for the priming site within the A1-Host *amt* gene sequence are labeled accordingly. Each sequence is written in 5' to 3' orientation.

Organism	Accession ID	AMT-557F priming site	AMT-715R priming site
		557 TTGTGCACATGACTGGCGGT 576	698 CTGGGTCGGCTGGTACG 715
A1-Host	MATOU-v1_26947674	TTGTGCACATGACTGGCGGT	CTGGGTCGGCTGGTACG
Chlamydomonas reinhardtii	XM_001697452.1	TCGTGCACATGACGGGTGGG	GTGGTTCGGCTGGTTCG
Chlamydomonas reinhardtii	XM_001693412.1	TCGTGCACATGACTGGTGGC	GTGGTTCGGCTGGTACG
Aureococcus anophagefferens	XM_009034951.1	TCGTGCACATGGTCGGCGGC	CTGGGTCGGCTGGTACG
Micromonas commoda	XM_002508364.1	TCGTGCACATGACCGGCGGT	CTGGGTCGGCTGGTATG
Emiliania huxleyi CCMP1516	XM_005785291.1	TGGTGCACTTGACAGGCGGC	CTGGATGGGCTGGTACG
Emiliania huxleyi CCMP1516	XM_005777257.1	TGGTGCACTTGACGGGTGGT	CTGGATGGGCTGGTACG
Monoraphidium neglectum	XM_014042203.1	TCGTGCACATGGTGGGCGGC	CTGGTTCGGCTGGTACG
Dunaliella viridis	GU592656.1	TTGTCCACATGACGGGTGGT	GTGGTTCGGCTGGTACG
Sorghum bicolor	JX294852.1	TCGTCCACATGGTCGGCGGC	CTGGTTCGGCTGGTTCG
Triticum aestivum	AY525637.3	TTGTCCACATGGTCGGCGGC	GTGGTTTGGCTGGTACG
Micromonas pusilla CCMP1545	GG663747.1	TCGTTCACATGGTCGGCGGG	CTGGGTTGGCTGGTACG
Ectocarpus siliculosus	FN648668.1	TCGTCCACATGACCGGCGGC	CTGGTTCGGATGGTACG
Ectocarpus siliculosus	FN648387.1	TCGTCCACATGACCGGCGGC	CTGGTTCGGATGGTACG
Ostreococcus tauri	CAID01000020.1	TCGTCCACATGACTGGTGGT	CTGGCTCGGCTGGTACG
Cylindrotheca fusiformis	AF360394.1	GAGC---CACTGCTCTTGTT	TTGGTTCGGATGGTATG
Chlamydomonas reinhardtii	DS496109.1	TCGTGCACATGACTGGTGGC	GTGGTTCGGCTGGTACG
Camellia sinensis	AB117640.1	TCGTACACATGGTCGGAGGC	ATGGTTCGGATGGTATG
Aureococcus anophagefferens	GL833121.1	TCGTGCACATGGTCGGCGGC	CTGGGTCGGCTGGTACG
Myxococcus xanthus	CP000113.1	TGGTGCACATGTCCGCGGGC	GTGGTTCGGCTGGTTCG
Micromonas sp. RCC299	CP001574.1	TCGTCCATATGACAGGCGGG	ATGGTTCGGTTGGTATG
Rhodothermus marinus	CP001807.1	TGGTGCATGCCGTCGGCGGC	CTTCTTCGGCTGGTTCG
Micromonas sp. RCC299	CP001576.1	TCGTGCACATGACCGGCGGT	CTGGGTCGGCTGGTATG
Perkinsus marinus	GG680729.1	TTGTCCATCTTACCGGAGGT	GTGGTTCGGATGGTTTG
Phaeodactylum tricornutum	CM000622.1	CAGTCCACATGACCGGAGGT	TTGGTTTGGCTGGTACG
Phaeodactylum tricornutum	CP001141.1	TAGTGCACATGACTGGAGGC	CTGGTTTGGTTGGTATG
Cylindrotheca fusiformis	AY651853.1	TTGTGCATATGTGCGGTGGA	TTGGTTCGGATGGTATG
Thalassiosira pseudonana	CM000639.1	CAGTTCACATGTGTGGAGGA	TTGGTTCGGATGGTATG

Supplementary Figure 3. Priming site of the newly designed AMT-330F/AMT-456R primers. The first row shows the priming site within the A1-Host *amt* gene. Following rows show the priming sites within the closely related amt sequences used to design the primers. Priming sites with gaps in the associated gene sequences are excluded from the figure. Mismatches in the gene sequences are shown in bolded black text. The end positions for the priming site within the A1-Host *amt* gene sequence are labeled accordingly. Each sequence is written in 5' to 3' orientation.

Organism	Accession ID	AMT-330F priming site (330–351)	AMT-456R priming site (437–456)
		**GTTCTTCCAGTTCGTCTTCGCT**	**CTTTCGTGTACCCTGTCGTC**
A1-Host	MATOU-v1_26947674	GTTCTTCCAGTTCGTCTTCGCT	CTTTCGTGTACCCTGTCGTC
Chlamydomonas reinhardtii	XM_001693412.1	GTTCTTCCAGTGGGCCTTCGCT	CCTTCGTCTACCCCGTCGTC
Aureococcus anophagefferens	XM_009034951.1	GTTCTTCCAGTTCGCCTTCGCC	GCTTCGTCTACCCCGTCGTC
Micromonas commoda	XM_002508364.1	CTTCTTCCAGTGGGCGTTCACC	GCTTCGTCTACCCCGTCGTC
Emiliania huxleyi CCMP1516	XM_005785291.1	GTTCTTCCAATATGTCTTCGCG	GCCTCATCTACCCCGTCGTT
Monoraphidium neglectum	XM_014042203.1	GTTCTTCCAGTGGGCGTTCGCC	CCTGGGTGTACCCTGTGGTG
Uncultured marine eukaryote	JN807524.1	GTTCTTCCAGTGGGCTTTCGCT	CTTTCATCTACCCCATCGTC
Uncultured marine eukaryote	JN807540.1	GTTCTTCCAGTGGGCATTCGCT	CTTTCATCTACCCCATCGTC
Dunaliella viridis	GU592656.1	CTTCTTCCAGTGGGCCTTCGCT	CCTTCGTGTACCCCGTGGTC
Tetraselmis chuii	JN807467.1	TATTCCAGTGGGCGTTTGCC	CCTTCGTCTACCCCGTCGTC
Sorghum bicolor	JX294852.1	CCTCTTCCAGTGGGCCTTCGCC	GCTTCGTGTACCCGGTCGTG
Triticum aestivum	AY525637.3	CCTCTTCCAGTGGGCCTTCGCC	GCTTCGTCTACCCTGTGGTG
Micromonas pusilla CCMP1545	GG663747.1	CCTCTTCCAATGGTCTTTCGCC	GCTTCGTCTACCCCACTGTC
Ectocarpus siliculosus	FN648668.1	GCTGTTCCAGTGGGCCTTCGCC	CGTTCATCTACCCCGTCGTC
Ectocarpus siliculosus	FN648387.1	GCTGTTCCAGTGGGCCTTCGCC	CGTTCATCTACCCCGTCGTC
Ostreococcus tauri	CAID01000020.1	CTTGTTCCAGTGGTCCTTCTCC	CATTCGTCTACCCGGTCGTC
Cylindrotheca fusiformis	AF360394.1	CTTTTTCCAATTCGCCTTTGCT	GTTTTGTCTATCCTGTCGTT
Chlamydomonas reinhardtii	DS496109.1	GTTCTTCCAGTGGGCCTTCGCT	CCTTCGTCTACCCCGTCGTC
Camellia sinensis	AB117640.1	CCTCTTCCAATGGGCTTTCGCC	GCTTCGTCTACCCAGTCGTA
Aureococcus anophagefferens	GL833121.1	GTTCTTCCAGTTCGCCTTCGCC	GCTTCGTCTACCCCGTCGTC
Myxococcus xanthus	CP000113.1	GCTGTTCCAGCTCAAGTTCGCC	TCGTCATCTACGCGCCGCTG
Micromonas sp. RCC299	CP001574.1	GCTGTTTCAATGGGCTTTCGCA	GTTTCATTTACCCTACTGTT
Rhodothermus marinus	CP001807.1	GCTGTTCCAGATGGTCTTTGCC	CCCTCATTTATCCGATCTAC
Micromonas sp. RCC299	CP001576.1	CTTCTTCCAGTGGGCGTTCACC	GCTTCGTCTACCCCGTCGTC
Perkinsus marinus	GG680729.1	GTTCTTCCAATGGGCTTTCTGT	CTTTTATCTACCCTGCGGTG
Phaeodactylum tricornutum	CM000622.1	GTTTTTCCAATTTGCCTTTGCT	GGTTTGTCTATCCAGTTGTC
Phaeodactylum tricornutum	CP001141.1	GTTGTTCCAGTACGCCTTTTCC	GATGGGTCTATCCGATTATC
Cylindrotheca fusiformis	AY651853.1	CTTCTTCCAATTTGCCTTTGCT	GTTTTGTCTATCCTGTCGTT
Thalassiosira pseudonana	CM000639.1	GTTCTTCCAATACGCCTTTGCG	GATTCGTCTACCCAGTCTGT

Supplementary Figure 4. Agarose gel electrophoresis image of PCR products generated for varied environmental samples using three newly designed primer pairs targeting the A1-Host *amt* gene. A dashed line separates the products amplified with the different primer pairs. Samples from different locations are indicated by numbers as follows: Santa Cruz Wharf (1), Baja California (2), Stn. ALOHA - HOT311 (3), Beaufort Sea (4), SIO Pier (5), Arctic 2016 (6), Tosa Bay (7), Coral Sea (8), and Stn. 17 – Gradients2 (9). The 100BP Ladder (Thermo Scientific GeneRuler 100bp Gene Ladder) and negative control are labeled L and – respectively.

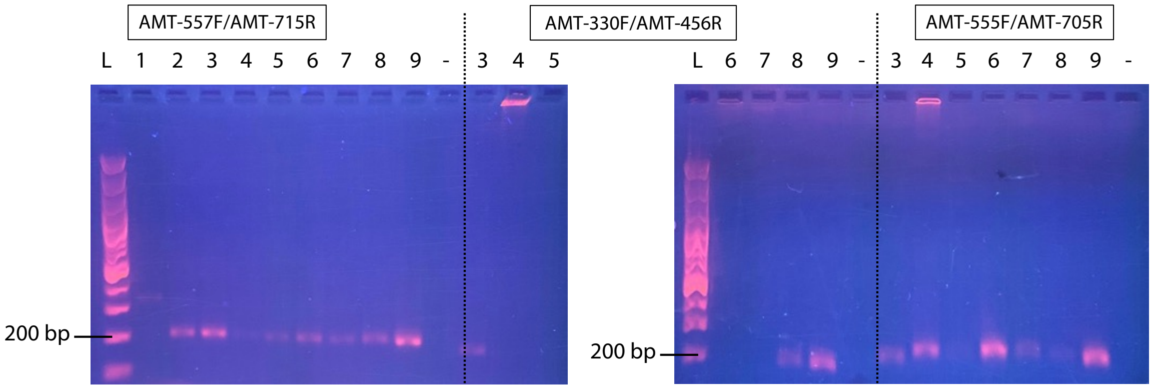

Supplement: Supplementary file 1 [file Data_Sheet_1.pdf]
